# Milk fat components with potential anticancer activity—a review

**DOI:** 10.1042/BSR20170705

**Published:** 2017-11-15

**Authors:** Luis M. Rodríguez-Alcalá, M. Pilar Castro-Gómez, Lígia L. Pimentel, Javier Fontecha

**Affiliations:** 1 Universidade Católica Portuguesa, CBQF - Centro de Biotecnologia e Química Fina – Laboratório Associado, Escola Superior de Biotecnologia, Rua Arquiteto Lobão Vital, Apartado 2511, Porto 4202-401, Portugal; 2 Research Center for Natural Resources and Sustainability (CIRENYS), Bernardo O’Higgins University, Fábrica N° 1990, Segundo Piso, Santiago de Chile, Chile; 3 Institute of Food Science Research, (CIAL, CSIC-UAM), Department of Bioactivity and Food Analysis, Food Lipid Biomarkers and Health Group, Campus of Autónoma University of Madrid, C/Nicolás Cabrera, Madrid 9. 28049, Spain

**Keywords:** ANTICANCER ACTIVITIES, Dairy products, fatty acids, phospholipids, sphingolipids

## Abstract

During many years, the milk fat has been unfairly undervalued due to its association with higher levels of cardiovascular diseases, dyslipidaemia or obesity, among others. However, currently, this relationship is being re-evaluated because some of the dairy lipid components have been attributed potential health benefits. Due to this, and based on the increasing incidence of cancer in our society, this review work aims to discuss the state of the art concerning scientific evidence of milk lipid components and reported anticancer properties. Results from the *in vitro* and *in vivo* experiments suggest that specific fatty acids (FA) (as butyric acid and conjugated linoleic acid (CLA), among others), phospholipids and sphingolipids from milk globule membrane are potential anticarcinogenic agents. However, their mechanism of action remains still unclear due to limited and inconsistent findings in human studies.

## Lipid metabolism, genetics and cancer

**Figure 1.**
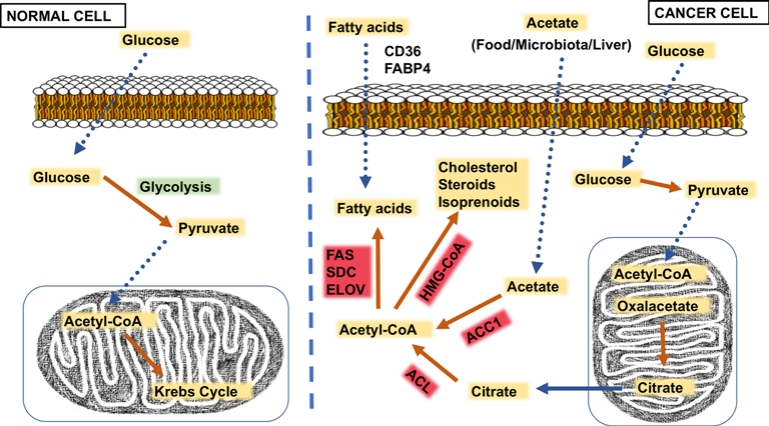
Metabolic alterations in cancer cells Yellow label: substrate/product; red label: enzyme; green label: reaction; dotted arrow: transport; orange arrow: reaction.

Cancer is the term to define a group of diseases characterized by uncontrolled growth and spread of cells affecting any part of the body. These ‘out-of-control’ cells also have the ability to invade surrounding lymph nodes, tissues or organs (metastatic cancer) as well as spread to distant sites in the body. This uncontrolled, oncogene-driven proliferation of cancer cells, lacking an efficient vascular system, quickly depletes the nutrient and oxygen supply from the normal vasculature and becomes hypoxic [1]. Due to this, one of the main hallmarks of cancer is a metabolic reprogramming consistent with the Warburg effect: increased glucose uptake and fermentation to lactate to promote growth, survival, proliferation and long-term maintenance [2]. Thus, normal cells use glycolysis to produce pyruvate that is transferred to the mitochondria to produce acetyl-CoA for further utilization in the tricarboxylic acid cycle (Figure 1), but cancer cells produce citrate that is converted in the cytoplasm into acetyl-CoA by the ATP citrate-lyase (ACL) [3].

Moreover, cancer cells can also rely on acetate uptake from three different sources: foods (e.g. meat, cheese, pickles), intestinal microbiota (fibre fermentation, besides resulting in short-chain fatty acid (SCFA), propionate and butyrate) and liver (while fasting, acyl­CoA thioesterase 12 (ACOT12) is activated and hydrolyses acetyl-­CoA) [4]. Thus, although membranes are passively permeable to acetic acid (p*K*_a_: 4.75), intestinal pH (5.5–7) favours anionic forms and therefore exists three active transport mechanisms involving: (i) monocarboxylate transporters (MCTs; coupling acetate plus SCFA uptake and excretion of bicarbonate), (ii) sodium-­coupled MCTs (SMCTs; that primarily uptake butyrate) and finally (iii) proton-coupled MCTs (co-transport of SCFA and H^+^) [4]. The latter form seems to be the main mechanism in colon and many other cancer types [5].

This scenario results in a characteristic lipogenic signature where acetyl-CoA serves as a substrate for acetyl-CoA carboxylase (ACC) to produce *de novo* synthesis of FA while other key enzymes of this pathway (i.e. fatty acid (FA) synthase (FAS); stearoyl-CoA desaturase (SDC); FA elongase 6/7 (ELOVL6/7); FA uptake as FABP4 and CD36) are up-regulated [6,7]. On the other hand, the mevalonate pathway is also involved and acetyl-CoA is used to produce cholesterol (CHOL), steroid hormones and non-steroid isoprenoids (needed for cell survival) by 3-hydroxy-3-methylglutaryl-coenzyme A reductase (HMG-CoA) [8]. Although many previous oncogenic studies concluded that enzymes of this pathway were unregulated [9], recent follow-up research investigations carried out with 1116 human patients reported that HMG-CoA expression was associated with less aggressive breast tumor characteristics [10].

There are two isoforms of ACC, one located in the cytosol (ACC1) and associated with the FA synthesis, while the second form is a negative regulator of β-oxidation as it allosterically inhibits carnitine palmitoyl transferase I (CPT1) [11]. Both are highly regulated by transcriptional factors as sterol regulatory element binding protein (SRBP1, regulating genes for FA and TAG synthesis; SRBP2, involved in CHOL biosynthesis) [12], liver X factors (activated by insulin to induce *SRBP1* mRNA) [13] and carbohydrate response element binding protein (ChREBP, activated by glucose to produce FA) [14]. Polymerization is required for the activity of these enzymes as mediated by MIG12 protein while ACC2 is also requires citrate [11,15]. However, Spot 14 (S14), a protein encoded by *Thrsp* gene, can form complexes with MIG12, therefore restraining the citrate-induced polymerization and acting as a metabolic inhibitor of ACC [16].

The hypoxic environment activates the transcriptional regulator hypoxia-inducible factor (HIF) by loss in hydroxylating capacity of oxygen sensors (i.e. PHD and FIH-1) or through an epigenetic way leading to both reduction in tumor-suppression functions (i.e. ING4, p53, PTEN, VHL) and activation of oncogenes (Ras, Raf, Src, mTOR and Myc) [17]. However, although HIF can provoke metabolic imbalance, there is also an HIF-independent pathway where it is activated by growth factors acting through PI3K/PTEN/AKT or RAS/RAF/MAPK signalling cascades [18].

Therefore, the key role of ACC and the fact that HIF up-regulates FAS and lipid transporters such as CD34 or FA-binding proteins [19] set a unique lipid signature in cancer cells: *de novo* synthesis directs towards production of palmitic and other unsaturated FAs as oleic acid together with lipid accumulation preferentially in the form of free FA (FFA), phospholipids (PLS) and cholesteryl esters [7,20]. According to this, research studies carried out both in breast cancer and normal adjacent tissues reported increments in the levels of phosphatidylcholine (PC), phosphatidylinositol (PI), phosphatidylethanolamine (PE) and sphingomyelin (SM), mainly in ER status, being negatively associated with the presence of PC (C16/C16) and PC (CN32) to survival [21]. These authors also observed that silencing of SCD, ACC, INSIG1 and ELOVL1 genes strongly decreased cell viability while knockdown of FAS increased apoptosis.

Lipid uptake is also an important feature as breast and liposarcoma cell lines produced CD36 (FA translocase) and lipoprotein lipase (LPL), the latter associated with an aggressive basal gene expression in breast cancer [22]. Furthermore, CD36 has been associated with activation of metastatic genes in cell lines and animal studies, while in humans it strongly correlates with poor disease free survival in lung, bladder, breast and melanoma cancer [23].

## Dairy fat and health

**Figure 2:**
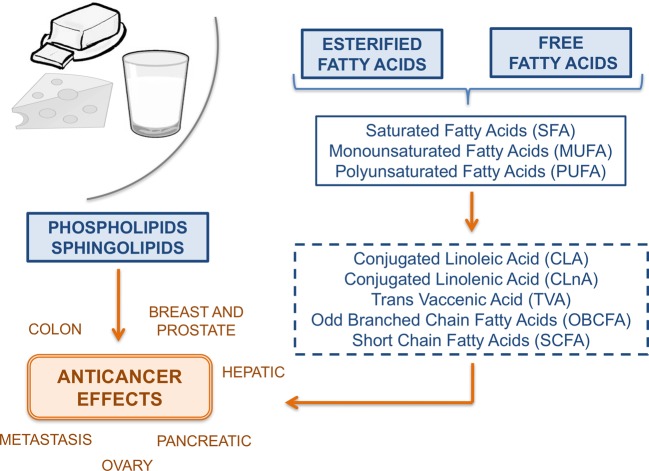
Schema summarizing the pathways related with the anticancer effects of milk lipids

Milk and dairy products are an important source of many essential nutrients as calcium, liposoluble vitamins (A, D, E and K) and carotenoids, bioactive peptides, essential FA, sphingolipids as well as other functional compounds with many benefits on health [24]. Despite this, during the last few years the dairy products intake has been perceived as unhealthy due to the presence of saturated FAs (SFA), *trans*-FAs (TFA) and CHOL as compounds associated with a higher risk of cardiovascular diseases, obesity or type 2 diabetes. Thus, some nutritional recommendations encouraged a low intake of fat dairy products or directly the consumption of low-fat dairy products, which means the loss of healthy components as some polyunsaturated FA (PUFA), vitamins or polar lipids (PL) [25]. Nowadays, the general perception about whole-fat dairy products has been improved by a high number of investigations which have not only refuted these ideas [26,27] but even highlighted the biological activity that some milk fat components can carry out in human health [28–30]. Supported by these studies, dairy fat is being re-evaluated and an increase in the interest of its components regarding to the beneficial functions in the maintenance, prevention and improvement of human health, as cancer, is occurring [31]. Cancer is a disease, whose incidence has been increasing in our society over the last years and, due to this, some reviews concerning this theme have been published. These studies have been focused on the effects of different anticancer compounds (Figure 2) but from non-dairy sources [24,32–34]. On the other hand, those carried out with dairy products do not include the lipid fraction and/or they are not aimed in anticancer activity [35–38].

Consequently, and overcoming this limited information, this review aims to expose the scientific evidence that relate the anticancer compounds in dairy fat, as FA, phospho- and sphingolipids and others related before.

## Dairy FAs and cancer

FAs in milk can be found esterified to the different compounds in fat (Table 1) as in triacylglycerides (TAG) and, to a lesser extent, to other glycerolipids as diacylglycerides (DAG) and monoacylglycerides (MAG) or PLS (phospho- and sphingolipids); they can also be found in free form (FFA). However, because of the animal origin of this fat, most of the FAs are saturated, reaching values from 60 to 70 g FA/100 g fat. On the other hand, the monounsaturated FA (MUFA) and PUFA contents comprise 20–25% and 3–5% respectively (Table 2). Among the SFA, it is remarkable that the presence of C14:0, C16:0 and C18:0, which in sum represents ~50% of total FA. Contrary to what has always been thought, in terms of their effects, it is known that in a scenario of moderate intake, there are no scientific evidence suggesting an increment in the risk of cardiovascular diseases [30,39,40]. Moreover, it has been reported that C18:0 has beneficial effects reducing the plasmatic CHOL. Other minor SFA are the SCFA C4:0 and C6:0 and the medium-chain FA (MCFA) C8:0 and C10:0 with different positive effects on the human health, not only as anticarcinogenics as it will be explained below, but also as antibacterial and antiviral agents [41–43]. Moreover, these latter groups of SCFA are easily absorbed from the intestine into the circulatory system without resynthesis of TAG and can be incorporated into tissues faster than FA with higher number of carbons, being very useful in case of gastrointestinal disorders [44] or used as a quick source of energy.

**Table 1:** Lipid composition of cows milk (minimum and maximum range in % of fat)

Compound	% minimum	% maximum
**TAG**	97.0	98.0
**DAG**	0.3	0.6
**MAG**	0.2	0.4
**FFA**	0.1	0.4
**Phospho- and sphingolipids**	0.2	1.0
**CHOL**	0.3	0.4
**Others**	Traces	

Data adapted from Jensen [59]. TAG: triacylglycerols; DAG: diacylglycerols; MAG: monoacylgliderols; FFA: Free fatty acids; CHOL: Cholesterol

**Table 2:** Mean composition (g/100 g of total FA) of main FA in cow, ewe and goat’s milks

FA	Cow	Ewe	Goat
**C4:0**	3.13	3.51	2.18
**C6:0**	1.94	2.90	2.39
**C8:0**	1.17	2.64	2.73
**C10:0**	2.48	7.82	9.97
**C12:0**	2.99	4.38	4.99
**C14:0**	10.38	10.43	9.81
**C14:1 *cis*-9**	1.08	0.28	0.18
**C15:0 *iso***	0.29	0.34	0.13
**C15:0 *anteiso***	0.50	0.47	0.21
**C15:0**	1.05	0.99	0.71
**C16:0 *iso***	0.22	0.21	0.24
**C16:0**	28.51	25.93	28.23
**C16:1 *cis*-9**	1.73	1.03	1.59
**C17:0 *iso***	0.55	0.53	0.35
**C17:0 *anteiso***	0.52	0.30	0.42
**C17:0**	0.73	0.63	0.72
**C18:0**	10.51	9.57	8.88
**C18:1 *cis*-9**	20.50	18.20	19.29
**C18:1 *trans* (total)**	4.25	2.90	2.12
**C18:2 *cis*-9 *cis*-12**	3.13	2.33	3.19
***C18:2 others***	1.03	0.88	0.70
**C18:3 *cis*-9 *cis*-12 *cis*-15**	0.59	0.63	0.42
**Conjugated linoleic acid (CLA)**	1.03	0.74	0.70
**Σ SFA**	64.97	70.65	71.96
**Σ MUFA**	27.56	22.41	23.18
**Σ PUFA**	5.78	4.58	5.01

Adapted from: Jensen [59], Alonso et al. [185], Goudjil et al. [186], Moate et al. [187] and Castro-Gómez et al. [200].

cis: cis double bond; iso/anteiso: branched chain fatty acid; trans: trans double bond; **Σ SFA**: total sum of saturated fatty acids; **Σ MUFA: total sum of monounsaturated fatty acids; Σ PUFA: total sum of polyunsaturated fatty acids.**

The MUFA fraction is mainly composed of oleic acid (C18:1 *cis* 9) associated with anticancer activities, reduction in plasmatic CHOL, improvement in the autoimmune system and reduction in the risk of inflammatory and cardiovascular diseases [45]. On the other hand, despite the reported negative effects of the octadecenoic TFA in the development of cardiovascular diseases [46,47], some studies suggest that dairy TFA can exert a different effect. Most of the studies, concerning these kind of FA, are carried out using industrial TFA from the biohydrogenation of vegetable oils (C18:1 *trans* 10, C18:1 *trans* 9) [48] while the main TFA in milk fat is *trans*-vaccenic acid (TVA) (C18:1 *trans* 11) (50–60% of total TFA and 2–6% of total FA) that may have positive effects on lipid metabolism and arteriosclerosis [49] as well as it is also the physiological precursor of rumenic acid (RA), a potent modulator of the lipid metabolism [50].

Related to PUFA profile, although they are in low concentration, there are interesting FA: C18:2 *cis* 9, *cis* 12 and C18:3 *cis* 9, *cis* 12 with not only beneficial effects for cardiovascular diseases but are also essential FA for the synthesis of other omega 6 and 3 compounds [51]. Furthermore, the different isomers of conjugated linoleic acid (CLA), overall RA C18:2 *cis* 9, *trans* 11, have been also attributed positive effects in cancer, diabetes, hypertension, immunology and body weight, among other beneficial effects [52–58].

### Anticancer effects

Some functional FA as SCFA (i.e. butyric acid, caproic acid and caprylic acid), odd-branched chain FA (OBCFA; e.g. margaric acid and phytanic acid), MUFA (e.g. palmitoleic acid, vaccenic acid, oleic acid) and PUFA (e.g. linoleic acid, linolenic acid, CLA and CLnA) [59,60] have been studied in many investigations due to the reported positive outcomes provided in different types of cancer risk.

#### CLA isomers

CLA is the acronym of CLA isomers (CLnA), mainly characterized by the presence of conjugated double bonds in the positions 6–14 (6,8; 7,9; 8,10; 9,11; 10,12; 11,13; 12,14), with four geometric groups (*trans,trans,cis,trans*–*trans,cis* and *cis,cis*) yielding 28 possible isomers [61]. Natural sources of these compounds are meat (0.12–0.68%) and mainly ruminants’ milk where they represent 0.34–1.07% of the total FA [62,63]. These isomers originated as a result of the biohydrogenation of PUFA (linoleic and linolenic acids from diet) by rumen bacteria and the action of the Δ^9^-desaturase enzyme (SDC) in the mammary gland on TVA (C18:1 t11), being RA (C18:2 c9, t11) the most abundant compound (75–90% of total CLA) [64]. These compounds attracted much attention since the studies of Ha et al. [65] describing the anticarcinogenic activities on mouse epidermal tumor of fried ground beef extracts, associating CLA with this activity. Although these properties are widely accepted as a result of several studies performed in cell lines and animals, there are few clinical studies conducted on humans which relate CLA intake with the incidence of cancer and most data are only from epidemiological studies [66]. Thus, cohort studies (4697 women, cancer free) during a follow-up period of 25 years and collecting food consumption data, reported a significant inverse association between milk intake and breast cancer [67]. Moreover, Aro et al. [68] after examining 403 Finnish patients during 4 years with breast cancer, reported that a CLA-rich diet (specially through cheese intake) might have anticarcinogenic effects in post-menopausal women. These results seem to be supported by further works involving 60708 women assaying the intake of high-fat dairy foods during 10 years. The authors concluded that the subjects consuming ≥4 servings of these foodstuffs (milk, cheese, sour cream and butter) showed lower risk of colorectal cancer while each increment of two servings reduced the risk in 13% [69]. However, these results are not conclusive since they were not specifically conducted testing CLA isomers but a food matrix. Furthermore, other studies carried out in women with breast cancer, invasive breast carcinoma or benign breast disease failed to reveal any positive correlation between CLA and incidence of cancer [70,71]. Nevertheless, it has to be mentioned that analysis of subcellular fractions from normal and cancerous parts of human tests revealed that CLA content was significantly higher in testicular carcinoma, but low in the mitochondrial fraction of this tumor in comparison with normal tissue [72].

Currently, two theories have been proposed to explain the possible biological effects of these isomers [73]. In the first one, CLA isomers replace arachidonic acid in the membrane phospholipids, altering the synthesis of eicosanoids that are involved in cell signalling. This may be the reason behind the increment in the levels of IgA, IgM and pro- (TNFα, cytokines and IL1β) and anti-inflammatory (IL-10) markers reported in both men (healthy) and women (healthy and overweight) assaying a dose of 1.1–3 g/day [54,74] or in human hepatitis B antibodies in men with 1.7 g/day for 12 weeks [75]. In a recent study in mice, C18:2 c9, t11 (RA) reduced arthritis severity equivalently to celecoxib, through a reduction in IL-6 and interleukin 1 β (IL-1β), thus suggesting that dietary RA may be an effective cyclooxygenase (COX2) inhibitor [76]. The second proposed mechanism places CLA as an agonist of all PPAR isoforms [77], proteins involved in adipocyte differentiation/proliferation, glucose uptake, mitochondrial function and inflammation [78].

In 2013, a clinical trial was assayed in women to determine whether CLA modulates the lipogenic pathway in human breast cancer tissue [79]. Results after intake of 7.5 g/d CLA (gel capsules containing 1:1 mixture of C18:2 c9, t11 and C18:2 t10, c12), showed reduced expression of both S14 and proliferative marker Ki-67 in primary invasive breast cancer. However, only patients with highest S14 IHC score (2) showed suppression, suggesting that initial metabolic status of these tumors may influence the CLA response. In further studies conducted on mice models of mammary cancer, animals lacking S14 resulted in the reduction in MCFA and FAS activity [80]. This impaired FA synthesis reduced solid tumor proliferation, cystic lesions and Src/Akt phosphorylation.

However, available information about anticancer properties of CLA on humans is still scarce. Therefore, there may be other yet unknown factors that are still challenges to address and more research focused on this topic is needed.

#### TVA

TVA is the main *trans*-octadecenoic FA in milk fat and its content comprises from 0.5–0.8 g/100 g fat in cow’s milk to 2 g/100 g fat in goat’s milk, although levels can be higher if the animal feed is supplemented with PUFA sources (e.g. extruded linseed) [81–83]. As commented above, TVA is produced by rumen bacteria during biohydrogenation of PUFA and metabolized in the mammary gland and other tissues into RA [84]. This bioconversion of TVA in humans would increase current estimates of CLA available for the general population by 6–10 fold. Thus, being precursor of an anticarcinogenic FA, some research works were focused in the positive effects of TVA on breast cancer. In Sprague–Dawley female rats injected with methylnitrosourea to induce mammary tumors and testing seven diets (*ad libitum*) with increasing content of TVA (0.13–1.60 g/100 g diet) and low in CLA (0.18–0.37 g/100 g diet), the results showed that the conversion of dietary TVA to RA caused a dose-dependent increase in the accumulation of CLA in the mammary fat pad and a parallel reduction in total tumor number and incidence [85]. This confirmed previous studies where the same effects were found and it was suggested that the anticancer response to TVA may be likely mediated by its endogenous conversion into CLA [86]. However, recently it has been reported that in cell lines T47D knockdown for SDC using siRNA, addition of TVA (50–200 μM) reduced proliferation [87].

It has been reported elsewhere that metabolic reprogramming is induced by long-term inflammatory signals in colorectal cancer cells [88]. Thus, adding elaidic acid (C18:1 t9; the main industrial *trans* fat) to umbilical vein endothelial cells (HUVEC) and human hepatocellular carcinoma (HepG2) cells increased SDC, while TVA reduced the tumour necrosis factor-α (TNF-α) induced gene expression of *TNF, VCAM-1* and *SOD2* in HUVEC and *IL-8* in HepG2 cells [89].

#### Conjugated linolenic acid isomers

CLA compounds are not the only conjugated FA that can be found in dairy fat. The presence of C18:3 c9,t11,c15 and C18:3 c9,t11,t15 has been described as the main conjugated linolenic acid isomers (CLnA) in milk fat and meat of ruminants as a result of the biohydrogenation processes in the rumen [90]. The concentration in milk for C18:3 c9,t11,c15 has been reported between 0.03 and 0.39 g/100g of fat and for C18:3 c9,t11,t15: 0.02–0.06 g/100g of fat [91,92]. Concerning ruminant meat, C18:3 c9,t11,c15 and C18:3 c9,t11,t15 isomers, were found in steer (0.08 and 0.02 mg/g respectively), cow (0.06 and 0.02 mg/g respectively) and goat (0.28 and 0.03 g/100 g fat respectively) [93–95]. These compounds have attracted much attention in the last years due to their biological effects similar to those exerted by CLA but at lower doses [96]. Currently, the investigations of anticancer properties have been carried out using vegetable oils with different isomer composition to that found in dairy products [97] and interestingly some results have shown that CLnA are metabolized to RA [98]. The CLnA isomers found in dairy products, C18:3 c9,t11,c15 and C18:3 c9,t11,t15, have a high bioactive potential as they combine in a same molecule conjugated double bonds and omega 3; thus, in order to bypass their low concentrations in those products that hinder the study of their bioactivity, some investigations assayed the microbiological production of these isomers [99] as well as the *in vivo* production [100]. This opens interesting possibilities for the future investigations of the anticancer activities of CLnA isomers present in new functional foodstuffs.

#### OBCFAs

Their structures are usually saturated acyl chains with odd number of carbon atoms and the branch is a methyl group in the –*iso* or –*anteiso* position, although there are also polybranched compounds. They can be found in most plants (trace levels), milk and adipose tissue in cows, goats and ewes and other animals with symbiotic fermentation [101]. The OBCFAs make up ~2% of total FA in cow’s milk and there are two possible sources for their presence: incorporation from rumen bacterial lipids or *de novo* synthesis. However, although odd chain and *anteiso* isomers can be synthesized in the mammary gland, the contribution of this process to milk content is negligible [102]. On the other hand, in bacterial synthesis, pentadecanoic (C15) and margaric (C17) acids are formed through elongation of propionate or valerate, whereas precursors of branched-chain FA (C13i, C14i, C15i, C16i, C17i, C18i, C13ai, C15ai, C17ai) are valine, leucine and isoleucine and their corresponding branched, short-chain carboxylic acids (isobutyric, isovaleric and 2-methyl butyric acids). The major branched-chain FA are the C15ai and C17ai that account for ~60% of total OBCFAs. Finally, the multibranched chain phytanic acid (3,7,11,15-tetramethylhexadecanoic) derived from phytol (side chain of chlorophyll) after released by rumen microorganisms is hydrogenated and oxidized into pristanic acid (2,6,10,14-tetramethylhexadecanoic) [101]. It has been described that rumen bacteria can also remove the α-carbon of palmitic (C16) or stearic (C18) acids to form the corresponding hydroxyl FA followed by further decarboxylation producing C15 and C17 [103]. Thus, these FA are taken up by the animal and used by the mammary glands to produce milk. Some issues have been pointed out according to the metabolism of phytanic acid in humans that after ingestion it is transported into peroxisomes but this FA can only be catabolized through α-oxidation to form pristanic acid, followed of isomerization by α-methylacyl-CoA racemase (AMACR), β-oxidation and finally production of CO_2_ and water in the mitochondria [104]. These multiple rounds of β-oxidation generate reactive oxygen species (ROS) with the potential to create molecular damage. Moreover, AMACR is strongly overexpressed in several cancers, notably in prostate and colorectal cancer and therefore some investigations were conducted to study the relationship between dietary intake, serum and tissue concentrations of phytanic acid and AMACR expression in histologically benign human prostate. Thus, analysis of plasma, tissues and food frequency questionnaire from men undergoing radical prostatectomy revealed that high-fat dairy intake correlated to circulating phytanic acid but not to its concentration in tissues and AMCR expression [105]. Investigations carried out with Finnish smokers showed that serum levels of phytanic and pristanic acids correlated with saturated fat, dairy products and butter but not with the risk of total or aggressive prostate cancer [106]. On the other hand, it has been reported elsewhere that phytanic acid is a retinoid-X receptor (RXR) and peroxisome proliferator activated receptor-α (PPAR-α) agonist at physiological levels and therefore acts on the energetic metabolism lipids [107]. Thus, OBCFA reduced the acetate incorporation into FFA and FA esters, showing the inhibition of FA biosynthesis (ACC down-regulation) and inhibition of NADPH production (through inhibition of glucose-6-phosphate dehydrogenase) in human breast cancer cells in a level comparable with that of CLA [108]. Recently, addition of C15i (35–140 µg/ml) to bladder cancer cells resulted in inhibition of proliferation in a dose- and time-dependent manner by inducing apoptosis through decreased expression of Bcl-2 and increased expression of Bax. This promoted mitochondrial dysfunction, leading to the release of cytochrome *c* from the mitochondria to the cytoplasm, as well as the proteolytic activation of caspases [109].

#### SCFA and MCFA

Butyric (C4), caproic (C6), caprilyc (C8) and capric (C10) FA are characteristic compounds of milk fat resulting from the fermentative processes of the rumen bacteria of the ruminants. These SCFA compounds are volatile and therefore responsible for the characteristic flavour in some products as cheeses. During cheese ripening, the action of lipases and esterases releases SCFA and MCFA [110]. Interestingly, the health value of goat milk has been recorded in ancient Jewish literature as well as in Asian and Mediterranean countries associated with the concentration of SCFA as C6, C8 and specially C10, which constitutes ~15% of the total FA content [111].

Some recent research works reported that MCFA produced from prebiotics reduced the risk of developing cancer [112] and highly ripened cheeses were capable of demonstrating antiproliferative activity and induction of apoptotic DNA damage on human leukaemia cells [113]. In further studies assaying the same cell line, these authors found that cheeses showed a concentration-dependent inhibition that may be also associated with the ripening length and therefore to the concentration of FFA [114]. Moreover, mixtures of C4, C6, C8 and C10 down-regulated genes involved in cycle division and progression of human cell lines of colorectal (cyclin-dependent kinase 2 (CDK2); cyclin-dependent kinase 4 (CDK4); CDC 28 protein kinase 1B (CKSIb); cyclin A2 (CCNA2) and cyclin D (CCND1)) and skin carcinoma (CKSIb, CCNA2 and CCND1) and mammary gland adenocarcinoma (CDK4, CKSIb, CCNA2 and CCND1) and, in general, lower the chain length lesser the efficiency [111]. This study also reported that for colon cancer cells the *Gadd45a* gene was up-regulated and incremented the caspase-8 activity. Other authors have pointed out that butyric acid (C4) is a potent therapeutic agent [115] but there are difficulties in reaching effective plasma concentrations *in vivo* while its prodrug (tributyrin (TB)) naturally present in some products (e.g. dairy products) has favourable pharmacokinetic properties and is better orally tolerated [116]. Accordingly, when male Wistar rats were fed a diet containing TB from maltodextrin and treated with N,N-Dimethylhydrazine to induce colon carcinogenesis, diet containing TB reduced the total number of aberrant crypt foci, increased the apoptotic index and reduced the DNA damage [117]. Moreover, in an experiment on induction of resistant hepatocyte carcinogenesis in male Wistar rats, gavage with TB (1 g.kg^−1^ body weight) reduced the number of GSTP-positive hepatic foci (a marker of liver carcinogenesis) together with genes involved in angiogenesis (Itgb6, Itgad, Ftl3, Map3k6, Mgp and Src) as it stimulates the vascular endothelial growth factor A (VEGFA) [118].

Acetylation/deacetylation of histone and non-histone proteins (e.g. p53, STAT, GATA) is one of the mechanisms related to transcription control [119,120]. Such processes have been also associated with cancer [121,122] where sirtuins (SIRT) have attracted much attention [123]. In recent investigations when hepatic cancer cells were exposed to butyrate, *miR-22* was expressed to inhibit SIRT-22 while up-regulating cytochrome *c*, caspase 9, caspase 3 and especially gsk-3 and PTEN [124]; the latter two are known to be cancer suppressor genes.

## Phospho- and sphingolipids

The PLS, which are classified in PE, PI, phosphatidylserine (PS) and PC, and the sphingolipids, mainly the SM, are PL with a crucial role of the maintenance and functionality of all the cell membranes [125,126]. Furthermore, they are not only critical for life maintenance; there are also numerous scientific evidence that support the positive effects of these dietary PLS on human health. Among these, remarkable are not only the anticancer effects, as will be further explained, but also those involved in cognitive development or neurological diseases (e.g. Alzheimer or Parkinson), fatty liver disease, reduction in cardiovascular risk and inflammation (rheumatoid arthritis) [32,33,35].

The PLS are widely distributed in foods due to their presence both in animal and vegetable cells. However, their composition is different according to source. Thus, while fruits, vegetables and tubers have almost non-existent content of PLS [127], egg yolk [128], soy [129], squid or krill oil [130] are the sources with high content of these compounds, providing mainly PC [33,131]. Ruminant brain tissues are a good source of all PL, although its consumption is strongly restricted due to the spongiform encephalopathy risk transmission. These limitations turn milk fat and specially the milk fat globule membrane (MFGM) in the only dietary source of all phospholipids and SM [132]. The MFGM surrounds TAG, protecting them against lipolysis and oxidation [133]. MFGM is a unique lipid trilayer where an inner monolayer, rich in protein and acquired from the endoplasmic reticulum of mammary cell, followed by a bilayer from the cell membrane resulting from the globule excretion [134]. The MFGM is mainly composed of proteins (50–70% weight) and PLS that are differently distributed throughout those membranes with a total content reaching 25–40% of total weight. The inner monolayer is mainly composed of PE, PI and PS, while the outer side of the MFGM bilayer contains mainly SM and PC. Interestingly, SM forms structures with CHOL (CHOL rafts) with critical roles in multiple cellular functions that include cell signalling, cell adhesion among others [135,136]. In terms of the concentration of each PLS in MFGM, PE, PC and SM are the most abundant moieties with content in the range 26.4–46.4%, 21.1–42.8% and 17.3–29.2% of total PLS respectively, while PI (3.4–14.1%) and PS (2.0–16.1%) are minor compounds (Table 3) [33,132].

**Table 3:** Phospho- and sphingolipid contents (% on total PLS) in cow’s milk

References	PE	PI	PS	PC	SM
Fagan and Wijesundera [188]	38.6	–	–	32.2	29.2
Avalli and Contarini [189]	32.3	9.3	10.5	27.3	20.5
Rombaut et al. [190]	33.2	5.2	9.3	27.4	25.1
Rombaut et al. [191]	46.4	5.3	7.4	21.1	19.8
Fong et al. [192]	32.6	7.6	5.3	33.2	21.3
Fauquant et al. [193]	36.4	7.6	6.5	32.1	17.3
Lopez et al. [194]	26.8	13.6	16.1	22.0	21.6
Sánchez-Juanes et al. [195]	28.5	14.1		32.7	23.0
Rodríguez-Alcalá and Fontecha [196]	38.5	6.5	7.7	25.9	21.4
Gallier et al. [197]	26.4	3.4	2.0	42.8	25.5
Le et al. [198]	36.9	6.1	6.3	27.0	23.7
Garcia et al. [199]	33.8	3.9	10.6	30.5	21.2
Castro-Gomez et al. [200]	42.0	3.9	3.4	29.3	21.0

Dates: Castro-Gomez et al. [33].

Other components present in low amounts in the MFGM are CHOL, FFA, glycoproteins and glycolipids [137].

### Anticancer effects

Many research investigations focused their studies in the determination of possible bioactivity of dietary PL through *in vivo* and *in vitro* assessments. Among all their positive effects [32,33,35], antiproliferative and preventive properties against cancer have been intensively studied. Despite most of them have been carried out testing PLS from egg, soy or marine sources, very limited information is available concerning dairy PL. According to the current literature, PLS bioactivity may rely on: (i) increased phospholipase A activity in cancer thus releasing bioactive milk FA [7], (ii) the reported effects of MFGM lipids to decrease CHOL and TAG levels [138] further supported by findings of lower levels of HMG-CoA (CHOL, steroid hormones and non-steroid isoprenoids) and SREBP1c (FA and TG synthesis) in human plasma [139] and (iii) SM properties are due to its metabolization from ceramides by sphingomyelinases that are activated by apoptotic signals and downstream proapoptotic members of the Bcl-2 family [140,141].

#### Colon cancer

Although there is a lack of investigation assaying milk PL, the existing bibliography allows hypothesizing the potential of such compounds. Thus, *in vitro* and *in vivo* studies carried out with soy and marine PLS showed antiproliferative effects in SW-480 and Caco-2 colon cancer cells, as well as an increment in the apoptosis in a colon tumor induced in F334 rats [142,143]. Some authors attributed the observed effect to the presence of omega 3 FA and improved bioactivity when they are linked to PLS rather than to TAG, due to better absorption [144–146]. However, this assumption is not valid for the reported antiproliferative effect observed with dairy PLS as they have low concentrations of omega 3 FA.

An *in vivo* study performed with 344 Fischer rats with aberrant foci crypts (colon cancer), showed that the diet supplemented with MFGM (rich in dairy PLS and bioactive proteins) and dairy fat (in proportion 1:1) at a concentration of 25 g/kg diet during 3 weeks reduced the incidence of this cancer [147]. This was also observed in other studies, which after isolating native MFGM from raw milk, reported inhibition of the proliferation of HT-29 and Caco-2 colon cancer cells *in vitro* [148,149]. These results suggest the use of MFGM and milk fat fraction as potential nutraceuticals or medical foods [150]. Based on these studies, the effects could be attributed to possible bioactive proteins present in MFGM and not the PL themselves. However a recent *in vitro* study [151] assessing the antiproliferative effect of a pure lipid concentrate of dairy PL, reported a total growth inhibition of colon cancer line HT29 at concentrations below 250 µl/ml. It is important to highlight the role of the use of food-grade solvent for PLS concentrates isolation, that seems to be a critical factor to the phospholipid and sphingolipid bioactivity maintenance [152].

Similarly, the role of SM in cellular growth control, differentiation, migration and apoptosis has led to an exhaustive study of this compound to be proposed as a possible treatment against colon cancer [153,154]. These results are supported by a research work in which it was observed that SM isolated from dairy fat decreased the number of aberrant foci crypts and provided a protective effect in induced colon cancer in mice ICR. They were fed *ad libitum* a diet consisting of dairy SM added at a concentration of 0.5 g/kg of food during 22 weeks [155]. This effect can be explained because dietary SM inhibited tumorigenesis and increased the alk-SMase activity. This enzyme is associated with the increase in the mRNA expression, which in turn, may contribute to the inhibitory effects of SM in this cancer. Interestingly, similar experiments in murines also fed with isolated dairy SM (as a supplement in the diet), showed that it was not only chemotherapeutic, but also chemopreventive when it is administered before the tumor induction [155–158]. These chemopreventive effects of SM appear to be due to its principal metabolites, i.e. sphingosine, sphingosine phosphate and ceramide, which induce apoptosis due to the modification of the expression of regulator genes in cancer [140,159]. Furthermore, a study by Schmelz et al. [160] reported that the administration of isolated dairy SM at a concentration of 0.005 g/100 g of diet in CF1 mice, transformed malignant adenocarcinoma to benign adenoma.

It is important to highlight that although SM seems to be the most active PL against cancer, other phospholipids also have an important role. Thus, it was reported elsewhere that milk SM is transported as a ceramide to lymph after hydrolysis and absorption; nevertheless intake of this sphingolipid together with other acylglicerols enhanced its bioavailability [161].

#### Breast and prostate cancer

Although studies *in vivo* or in humans have not been carried out with breast and prostate cancers, these carcinogenic cells have been widely studied and nowadays it is well known that prostate and breast cancer cells are particularly rich in CHOL rafts, whose density modifies cellular functionality and the evolution of metastasis [162].

The sphingolipids in the cell membranes are not the only PL with importance in breast and prostate cancer development. Indeed, the presence or changes in some PLS seem to be an important biomarker. The study of Doria et al. [163] concerning breast cancer cells MCF10A, T47-D and MDA-MB-231 showed different PL composition in the membrane during the development of the disease. In the same way, three PC molecular species were also reported as biomarkers of progression in prostate cancer cells [164]. These findings need further confirmation due to the importance to understand the new metabolic routes involved in disease progression, early diagnosis or even for the development of new treatments.

In terms of dairy products intake and its relation with breast and prostate cancer, on one hand, Dong et al. [165] found that the increase in the consumption of dairy products may be associated with a reduced risk of breast cancer, however this effect is not attributed to dairy PL specifically. On the other hand, Parodi [166] did not find a correlation between dairy products intake and prostate cancer.

Related to the use of PL on breast and prostate cancer cells, a study of Abd El Baky et al. [167] reported an antiproliferative effect of microalgae PLS on MCF-7 breast cancer cells. The same result could not be concluded in a recent study [151] carried out with dairy PLS in the same cell line which did not report anticancer activity in an *in vitro* experiment. This difference could rely on the different lipid isolation methods used or on the different composition of the assessed samples.

#### Hepatic cancer

The liver cells have the peculiarity of easily incorporating omega 3 FA, which facilitates the lipogenesis inhibition and induces the apoptosis [168].

Although there is a lack of studies assaying dairy PLS against hepatic cancer, it is remarkable the effect of PL from other sources against this type of cancer. A cell growth decrease by 50% was observed in hepatic cancer cells Hep-G2 in a recent study using PL from microalgae [167]. Furthermore, other *in vitro* studies, using the cells Hep-3B, Hep-G2, HuH-7 and Alexander observed that after their treatment with isolated PC from soy and egg yolk, the cancer cell growth was inhibited. *In vivo* experiments carried out with the same cells and egg PL concentrate in Sprague–Dawley rats, also showed the same anticancer effects, which was potentiated by the presence of menaquinone-4 (vitamin K_2_). These rats were intragastrically fed a diet enriched at concentrations of 0.05 g of PL concentrate/100 g of diet during 14 weeks [169,170].

Moreover, sphingolipids also provide beneficial effects against hepatocellular carcinoma, even when external treatment is not administered: it has been suggested that sphingolipid metabolic pathway (e.g. conversion into ceramides) may be implicated [154].

#### Pancreatic *cancer*

The effects of PL in pancreatic cancer are mainly related to sphingolipids. Although *in vivo* or human studies have not been reported, it is well known that during the treatment with radio- and/or chemotherapy, there is a natural increment in plasma sphingolipids resulting in an improvement in the treatment effect. A possible explanation is that when the two proinflammatory cytokines, tumour necrosis factor-α (TNF-α) and IL-1β are present in blood, they play an important role in hydrolytic generation and accumulation of sphingolipids [171,172]. These latter compounds play a critical role in apoptosis according to their action as second messenger in the activation of enzymes involved in this process; pharmacologic manipulation of intracellular ceramide levels leading to attenuation or enhancement of drug resistance [173]. This is supported by a study in which utilization of SM isolated from egg yolk on pancreatic cancer cells AsPc1, increased the chemotherapeutic 5-fluorouracil effect [174].

#### Ovary *cancer*

It seems to exist in a relationship between the anticarcinogenic effect of ceramides in pancreatic and ovary cancer. A study carried out *in vitro* with drug-resistant ovary cancer cells (SKOV3), found evidence of inhibitory effects and apoptosis signalling when a synthetic ceramide was combined with the anticancer drug paclitaxel [175]. *In vivo* experiments were also conducted in female nu/nu (athymic) mice inoculated with SKOV3 tumor. The intravenous administration of the ceramides with the anticancer drug paclitaxel at a concentration of 80 mg/kg of rat and 20 mg/kg of rat respectively, resulted in a higher diminution of the tumors than when only the chemotherapeutic drug was used [176].

However, research studies focused on the effects of dairy PL on ovary cancer cells are limited. Only one study reported that the administration *in vitro* of a concentrate of phospho- and sphingolipids from buttermilk showed antiproliferative effect on ovary cancer line NCI/ADR-RES, at a concentration of ~100 µg/ml. The same research work found that IC_50_ in this cancer cell line of a dairy fat was 100 µg/ml. This activity could be explained by the presence of ~5% of SM in the extract [151].

### Metastasis

Some investigations suggest that PL may affect cancer cells migration. A meta-analysis by Sun et al. [177] concluded that dairy products consumption was not associated with gastric cancer but regarding metastasis, PL seem to have beneficial properties. The phospholipids PE and PC and SM, exerted positive effects in gastric cancer cells NUGC-4 metastasis as it reduced adhesion and migration to other tissues [178].

Lysoform structures (phospholipids lacking one FA), e.g. lyso-PC, have also been shown to possess chemopreventive and antimetastatic properties. It occurs because when the PL release the FA, the lysoforms make cancer cells lose the adhesion capacity to other cells and platelets, inhibiting the migration to other tissues. This was supported by a study [179] which demonstrated that the commercial lyso-PC reduced B16-F10 melanoma cell adhesion, mediated by the expression of VLA-4 and P-selectineto in *in vitro* experiments. Furthermore, the same study in male C57B1/6N mice with the same intravenous cancer cells for lung invasion, observed that administration of lyso-PC at 450 mmol/l reduced the cell dissemination [179]. Despite the beneficial results against the metastasis, further studies are needed to gain deeper insight into the molecular mechanisms of lyso-PC.

### Other types of cancer and the role of dairy PL in the treatment

Other investigations studied the effects of phospho- and sphingolipids from dairy sources against different cancer types. However, they reported non-conclusive results and therefore more research studies are needed. As an example, a recent research work associated for the first time antiproliferative activity of dairy PL against kidney and leukemia cancer cells [151]. These authors reported an IC_50_ on cell lines 786-O (kidney/adenocarcinoma) and K562 (bone marrow/myeloid leukaemia) at concentrations of 100 µg/ml and TGI at concentrations over 250 µg/ml when assaying a PL enriched fraction from buttermilk. Furthermore, Russell et al. [180] suggested that milk phospholipids act in a protective manner against UV exposure which is directly related with the development of skin cancer. Inspite of this, evaluation of the DNA damage is essential to assess if UV exposure alters the protein regulation within the cells.

Another example is bladder cancer. In a meta-analysis study [181] authors revealed an inverse association between whole milk intake and bladder cancer risk. Although consumption of skimmed milk was associated with development of bladder cancer [182], these effects were mainly attributed to caseins.

In terms of PL functionality, milk phospholipids have been also assessed in order to produce liposome structures. When assaying carriers for anticancer etoposide, PL isolated from camel milk showed highest efficiency delivering this drug in a mouse model of fibrosarcoma [183]. Recently, milk-derived exosomes, biological nanovesicles that are involved in cell–cell communication have been isolated and described in bovine milk. These exosomes can act as carriers for chemotherapeutic/chemopreventive agents [184].

## Conclusion

Milk fat not only provides beneficial compounds for human nutrition, but also interesting activity against different kinds of cancer. This health condition is a group of disorders characterized by a profound metabolic reprogramming to sustain the cell proliferation partially relying on the FA, PL and cholesteryl ester synthesis as well as lipids uptake.

Current bibliography highlights dairy FA, namely butyric and some other short and MCFA, CLA and CLnA, TVA, branched-FA as well as different phospho- and sphingolipids as promising anticancer molecules. FA bioactivity is mediated by down-regulation of the ACC, FASN and HMG-CoA or specific genes associated with cell proliferation and apoptosis. About PL, the positive effects may be related to action of phospholipase, therefore releasing FA, reduction in adhesion capacity (metastasis) or through transformation of SM on to ceramides to downstream apoptotic Blc-2 proteins.

Although many research studies have pointed interesting properties against different forms of this disease, *in vitro* and *in vivo* studies results remain nowadays inconclusive and without a clear pharmaceutical application.

This clearly shows that more research is still needed involving human clinical trials allowing a better understanding of anticancer biochemistry related with fat dairy compounds.

## Abbreviations

ACC: acetyl-CoA carboxylase
AMACR: α-methylacyl-CoA racemase
CCNA2: cyclin A2
CCND1: cyclin D
CDK4: cyclin-dependent kinase 4
CHOL: cholesterol
CKSIb: CDC 28 protein kinase 1B
CLA: conjugated linoleic acid
CLnA: CLA isomer
FA: fatty acid
FAS: FA synthase
FFA: free FA
HIF: hypoxia-inducible factor
HMG-CoA: 3-hydroxy-3-methylglutaryl-coenzyme A reductase
HUVEC: umbilical vein endothelial cell
IL-1β: interleukin 1 β
MCFA: medium-chain FA
MCT: monocarboxylate transporter
MFGM: milk fat globule membrane
MUFA: monounsaturated FA
OBCFA: odd-branched chain FA
PC: phosphatidylcholine
PE: phosphatidylethanolamine
PI: phosphatidylinositol
PL: polar lipid
PS: phosphatidylserine
PUFA: polyunsaturated FA
RA: rumenic acid
SCFA: short-chain FA
SDC: stearoyl-CoA desaturase
SIRT: sirtuin
SM: sphingomyelin
SRBP1: sterol regulatory element binding protein
S14: Spot14
TAG: triacylglicerol
TB: tributyrin
TFA: *trans*-FA
TNF-α: tumour necrosis factor-α
TVA: *trans*-vaccenic acid
